# Improving the filtration properties for manganese tetroxide mud utilizing perlite particles to drill wide-range permeability sandstone formation

**DOI:** 10.1038/s41598-022-21897-8

**Published:** 2022-11-02

**Authors:** Jaber B. Al Jaberi, Badr Bageri, Salaheldin Elkatatny

**Affiliations:** 1 Department of Petroleum Engineering, King Fahd University of Petroleum & Minerals, Dhahran, Saudi Arabia; 2 Center for Integrative Petroleum Research, King Fahd University of Petroleum & Minerals, Dhahran, Saudi Arabia

**Keywords:** Geochemistry, Mineralogy, Petrology

## Abstract

A required feature of any drilling formulation is to mitigate the formation damage by having an excellent filtration and filter cake properties. The key factor for preventing and limiting formation damage is to improve the sealing qualities of the planned drilling fluid. In this study, a new novel filtration agent called “perlite” was introduced for improving the manganese tetroxide mud cake ability for better sealing features. The perlite particles were loaded to formulation containing the manganese tetroxide as weighting agent. The water-based drilling mud was designed at high densities (14.25 and 17.2 ppg). Perlite was added in varied concentrations to reach the optimum performance. The filtration test conducted at reservoir temperature of 250 °F and a differential pressure of 300 psi to form the filter cake. The tests were performed using sandstone cores with two different permeability categories (low and high permeabilities) as the filtration media. This gave the full picture of perlite performance as implemented for different formation properties and considering the drilling fluid properties. The formed filter cake structure and chemical composition was evaluated using scanning electron energy-dispersive X-ray (SEM–EDS). The presented results illustrated how the perlite was compatible to be added to the manganese tetroxide weighting agents in the same formulation. In addition, it has the capacity to improve the filter cake's sealing qualities, lowering the filtration volume by 41% and the filter cake internal and external layers permeability by 58% and 25%, respectively. Moreover, the EDS analysis showed that the perlite particles are concentrated generally in the internal layer of the filter cake.

## Introduction

Optimizing the drilling fluid formulation is a critical in the oil and gas industry since it considered as the blood for drilling operation^1^. The optimization includes selecting the correct additives with the correct amount to drill the well efficiently and economically. These additives provide many functions including controlling the formation pressure, cleaning the wellbore and carrying the cutting, forming a filter cake to protect the formation, etc.^2,3^. Depending on the well depth, formation type and feasibility, the need of these additives change^4^. Scientists and researchers proposed many additives over the years, for instance, using durian rind as a lost circulation material^5^, utilizing different classes of ion liquid to improve the mud rheology^6,7^, implementing polypropylene and polyethylene beads to improve the cutting transport efficiency^8–12^, employing Nano-material to enhance the drilling fluid properties^13–15^, etc. One of the vital additives is the weighting material which provide the needed density in the mud formulation^16^. The amount of the weighting material needed is changing based on many factors, for example, drilling deep well require high density to control the formation pressure which translate to high dosage of the weighting material depending on the weighting material specific gravity^17^. Moreover, they impact the other properties of the mud including the rheology, filtration behavior and filter cake. Hence, it is important to select the appropriate weighting material in the mud formulation.

Barite, hematite, ilmenite, gelana, and other weighing minerals are utilized in the oil and gas sector^18–24^. Furthermore, some of these materials were micronized to improve their properties and reduced related issues such as sagging^17,25–28^. Manganese tetroxide (Mn_3_O_4_) was proposed to be used as weighting material, it has a specific gravity of ~ 4.8 g/cm^3^, low particle size around 1 μm, and spherical shape^29,30^. These features promoted the Mn_3_O_4_ (Micromax) as good candidate for drilling and completion fluids^31^. Al-Yami et al.^32^ formulate and investigated water-based formulation composed of KCl/Mn_3_O_4_, and compared it to two of the existing formulations (i.e., KCl/BaSO_4_/CaCO_3_ and CHKO_2_/CaCO_3_). They found that Micromax formulation showed better thermal stability compared to the other formulations with better filtration and rheological properties. Moroni et al.^31^ used Mn_3_O_4_ as weighting agent in invert emulsion for completion fluid. They showed two field cases in UK where they needed to bargain between using the brine to provide high density and by using oil to stabilize shale formation. In the first case, they needed 1.95 S.G. completion fluid to run a completion string. They used Mn_3_O_4_ as weighting material in mineral oil invert emulsion with the focus in the lab was the sag properties over 14 days and the film characteristics on the drilling tools. The results in the lab were excellent which led to successful implementation in the field. The second case was in directional well with borehole stability issue related to the sand production bounded by clay/shale formations. The proposed solution was to install sand screens, to achieved that a solid free non-aqueous fluid was required. An invert emulsion was used and Mn_3_O_4_ was added to reach the required density (i.e., 1.44 S.G.). The used completion fluid helped positively in the installation process of the sand screens. Moreover, Micromax can help in minimizing the sagging in other drilling fluid such as barite. Basfar et al.^33^ solved barite sagging issue in invert emulsion fluid by using mixture of barite and Micromax. They studied two different percentages of Micromax including 15 wt% and 30 wt%, they found 30 wt% is the optimum percentage that eliminated the barite sagging. Also, the other drilling fluid properties were enhanced including the filtration, rheology and viscoelasticity. Also, they investigated the same solution for barite sagging in water-based drilling fluid^34^. Their results showed that 25 wt% was able to eliminate the barite sagging and enhance the drilling fluid's overall qualities.

The influence of the weighting components in the drilling formulation on filtration and filter cake characteristics is also important. Al-Jaberi et al.^35^ studied how the weighting materials can impact several drilling fluid properties. In comparison to the filtration medium characteristics, they discovered that the weighting material is the most important influence in filtration behavior and filter cake features. In addition, the weighing material account for almost 70–90% of the filter cake mineralogy^19^. Furthermore, the weighting material might damage the formation during the formation and removal process of the filter cake^36,37^, hence it is critical to add filtration agents to minimize the filtration to the formation. Many filtering agents have been developed and employed in the oil and gas sector throughout the years and despite the success stories for them. The researchers are still anxious to improve the existing ones and recommended new ones that are superior in performance, economical, and environment friendly. Perlite is an amorphous volcanic mineral that has the ability to expand 20 times it is original with high absorption of water. It is used in a variety of sectors, including agriculture, construction, and thermal insulation, etc. It was also utilized as a drilling fluid and cement additive in the oil and gas sector. It showed the ability to minimize the weighting material sagging, improve the rheological properties, and enhance the filtration and the filter cake properties for different drilling formulations^38–40^. Furthermore, the cement properties including the compressive strength, young modules, and viscosity were improved with the addition of perlite particles^41–43^.

In this work, perlite was implemented as filtration agent for improving the filtration properties of Micromax water-based drilling fluid. Two different dosages of Micromax were applied in preparing the drilling fluid for this work that accordingly produced two densities equal to 14.25 and 17.2 ppg. The perlite was loaded in different concentrations and sandstone core samples with various permeability were employed as filtering media. The investigation covered the filtration behavior and the filter cake properties (i.e., thickness, permeability, porosity).

## Materials

**Figure 1:**
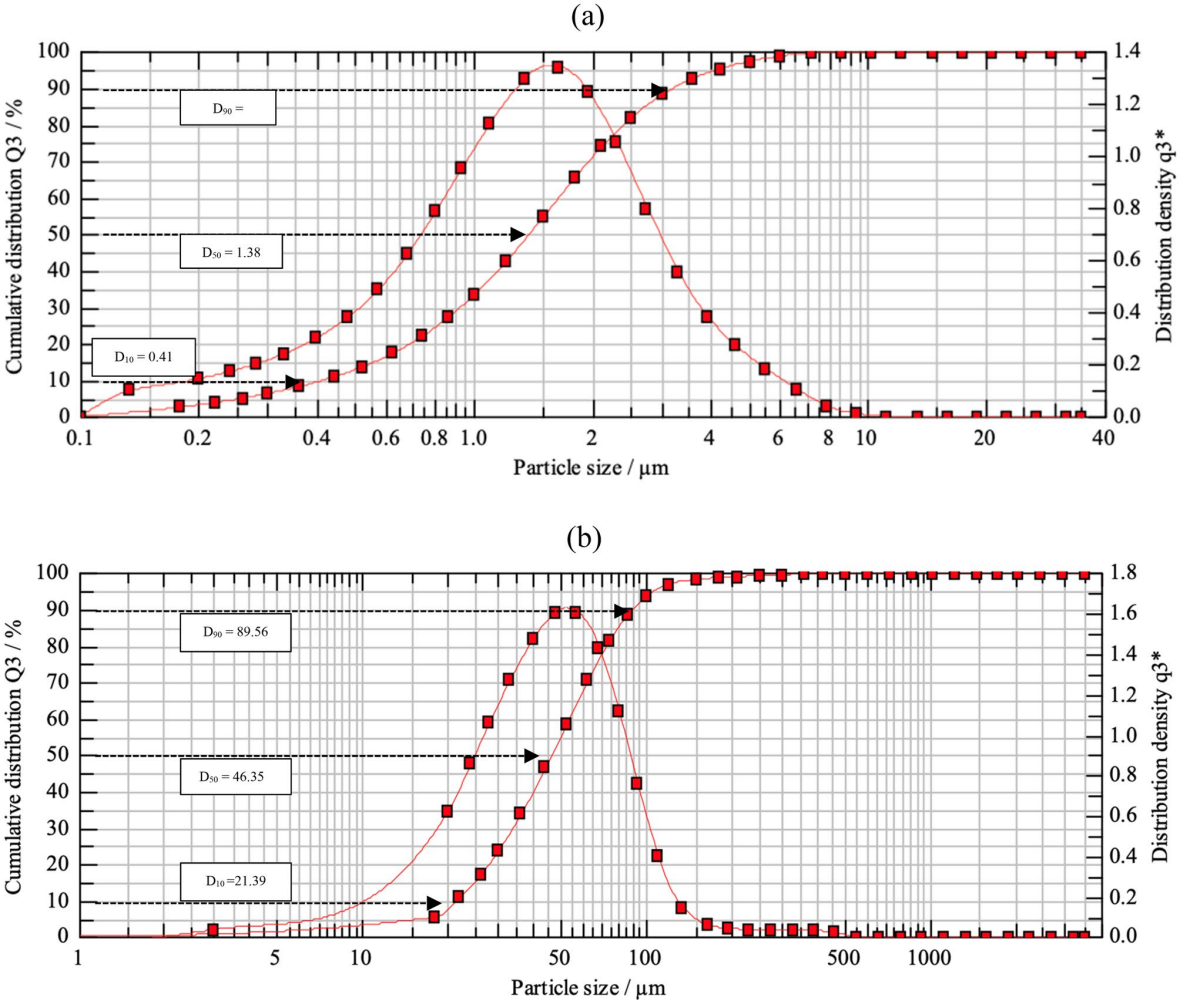
PDS for (**a**) Micromax and (**b**) perlite.

**Figure 2:**
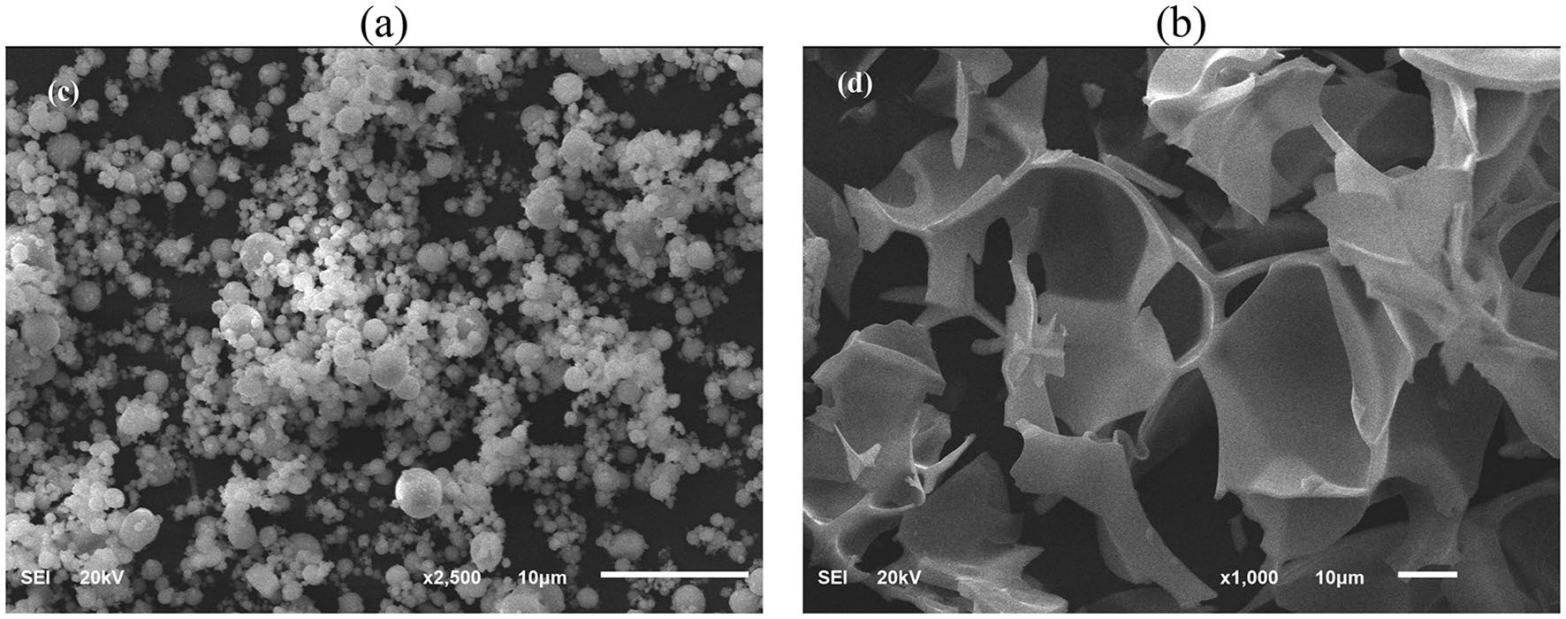
SEM for (**a**) Micromax and (**b**) perlite at 10 µm.

Several materials were used in this work to formulate the drilling fluid, but the focus in this work on Micromax and perlite which were characterized. As stated previously, perlite is a material that forms by the obsidian hydration process and it consists mainly of silicon. Manganese tetroxide (Mn_3_O_4_) was used as weighting material in this work. The energy dispersive X-ray spectroscopy (EDS) results for perlite and Micromax is shown in Table 1. The used Micromax contain three main elements represented by carbon, oxygen and manganese. The particle size distributions for both Mn_3_O_4_ and perlite are shown in Fig. 1, the D_10_, D_50_, and D_90_ for perlite are 21.39 μm, 46.35 μm, and 89.56 μm respectively. For the Micromax, the D_10_, D_50_, and D_90_ are equal to 0.41 μm, 1.38 μm, and 3.23 μm, respectively. Clearly, the particle size of the perlite is larger in comparison to the perlite particles. Furthermore, Fig. 2 shows the morphology of the Micromax and perlite particles using SEM images. Micromax has spherical shape while the perlite exhibits a fragment shape.

**Table 1:** EDS results for (a) Micromax and (b) perlite.

(a)				(b)						
	C	O	Mn		O	C	Si	Al	Na	K
Wt.%	40.7	31.0	28.3	Wt.%	48.1	25.0	19.1	4.1	2.0	1.7
σ	1.0	0.9	0.6	σ	2.1	3.0	0.9	0.3	0.2	0.2

Six drilling formulations were prepared in this work, they consist of several additives with specific function per each. Water used as a continuous phase and defoamer to inhibit the form of foams. Sodium carbonate function is to keep the calcium concentration within the acceptable range, and Xanthan gum polymer (XC-polymer) used as a viscosifier. Starch (i.e., potato type) and polyanionic cellulose (PAC) were used as filtration agents. Bentonite (i.e., sodium based) is a clay that dispersed to raise the viscosity. As a pH controller, clay stabilizer, and bridging material, potassium hydroxide, potassium chloride, and calcium carbonate were utilized, respectively. Micromax was added as weighting material, it was loaded with two different dosages including 350 g for sample 1–4 and 250 g for sample 5 and 6. Perlite concentration was varied from 0 to 3 to assess its impact on the filtration properties. They were labeled as MM14-P0, MM14-P3 MM17-P0, MM17-P1, MM17-P2, and MM17-P3where the first number represent the density in pound per gallon (ppg) and the second number show the perlite concentration in gram (gm). The entire formulation with ordered used are displayed in Table 2.

**Table 2:** Drilling fluid formulation.

	MM17-P0	MM17-P1	MM17-P2	MM17-P3	MM14-P0	MM14-P3
Density, ppg	17.20				14.25	
*Water,* bbl	245					
*Defoarmers,*	0.1					
*Na*_*2*_*CO*_*3*_	0.5					
*XC-Polymer,* lb	0.5					
*Starch,* lb	6					
*Bentonite,* lb	4					
*Perlite,* lb	–	1	2	3	0	3
*KOH,* lb	0.5					
*PAC,* lb	1					
*KCl,*lb	20					
*CaCO*_*3*_* (50 micron),*lb	5					
*MicroMax,* lb	350				250	

Berea sandstone was used as filtration medium, four sister cores were used for mud formulation (MM17-P0, MM17-P1, MM17-P2, and MM17-P3), different two sister cores were used for the remaining mud formulation samples (MM14-P0 and MM14-P3). Their porosity and permeability were measured using porosimeter and prop permeameter as demonstrated in Table 3.

**Table 3:** The mud formulation, and the corresponding core samples properties.

Formulation	Perlite Conc. (lb)	Porosity %	Permeability, md	Permeability Classification
MM17-P0	0	17.24	28.53	Low
MM17-P1	1	16.97	30.01	
MM17-P2	2	17.68	33.68	
MM17-P3	3	19.43	40.67	
MM14-P0	0	23.32	408.23	High
MM14-P3	3	21.54	435.37	

## Methods

To make the filter cake, the filtration tests were carried out using a high-pressure high-temperature filter press (HPHT filter press). The tests were conducted with conditions of differential pressure equal to 300 psi and temperature fixed at 250 °F. The filter cakes were formed over the face of sandstone cores. After that, the filter cake measurements were taken including the weights and the dimension. The filter cake was dried inside a vacuum oven at temperature of 180 °F. The cake was coated with gold and placed inside the SEM to analyze the chemical composition.

## Results

**Figure 3:**
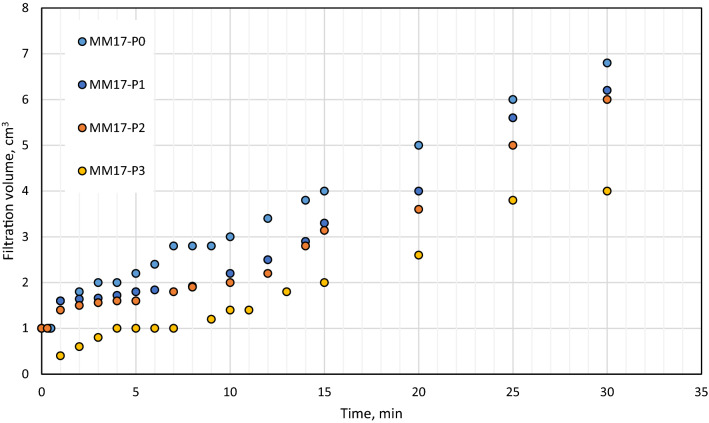
Filtration behavior as function of perlite concentration for samples with mud density equal to 17.2 ppg.

**Figure 4:**
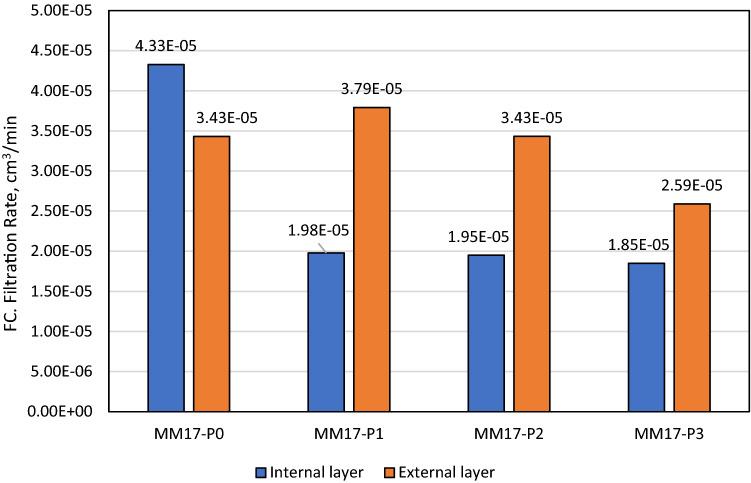
Filter cake rate for internal and external layers as function of perlite concentration for mud density equal to 17.2 ppg.

**Figure 5:**
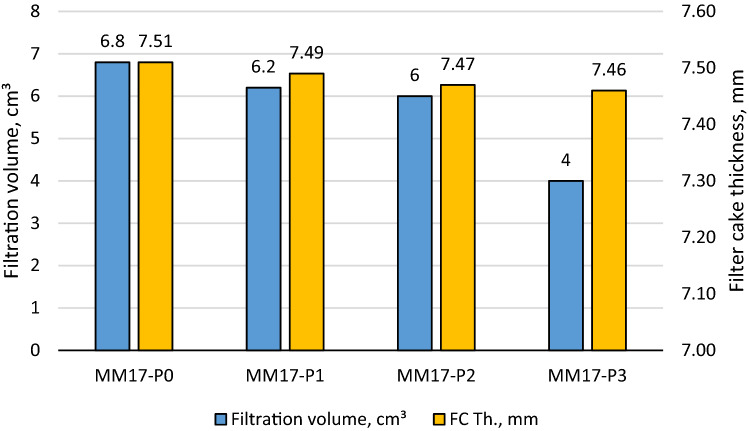
Filter cake thickness and filtration volume as function of perlite for mud density equal to 17.2 ppg.

As stated previously, perlite showed excellent features as filtration agent in barite and hematite drilling fluids. With a concentration of 2 lb/bbl as the optimal concentration, it was able to reduce barite drilling fluid filtration volume by 40% and filter cake thickness by 30%^38^. The optimal concentration in hematite-based drilling fluid was 4 lb/bbl, which reduced filtering volume and filter cake thickness by 50% and 49%, respectively^39^. The filtration behavior for Micromax drilling fluids tested in this work with density of 17.2 ppg (i.e., MM17-P0, MM17-P1, MM17-P2, MM17-P3) are shown in Fig. 3. Based on the figure, the 7 min marks the transition from the internal layer to external of the filter cake. This is indicted by the change of the filter cake rate before and after the 7 min. From this figure, the buildup rate for both layers and the filtration volume can be determined which is plotted in Fig. 4. The buildup rate is simply calculated based on the slope in each region. Initially, it is obvious that the perlite controlled the buildup rate of both layers. MM17-P0 showed the highest buildup rate in the filter cake internal layer compared to other samples. The filter cake buildup rate for MM17-P0 is 4.33E−5 cm^3^/min compared to 1.98E−5, 1.95E−5, and 1.85E05 for MM17-P1, samples-3 and MM17-P3, respectively. The spurt loss was same for the first three samples, but it decreased greatly in MM17-P3 due to the perlite high concentration. The external layer was less effected by the perlite particles, MM17-P3 showed the lowest building rate which equal to 2.59E−5 cm^3^/min compared to 3.43E−5, 3.79E−5, and 3.43E−5 for MM17-P0, MM17-P1, and MM17-P2, respectively. Similar trend to the internal filter cake buildup rate can be observed in the filtration volume, as the perlite concentration increased the filtration volume decreased as shown in Fig. 5. MM17-P0 had filtration volume equal to 6.8 cm^3^ compared to 6.2, 6, and 4 cm^3^ for MM17-P1, MM17-P2, and MM17-P3, respectively. Perlite had essentially little effect on filter cake thickness, with all samples having a value close to 7.5 mm.

**Figure 6:**
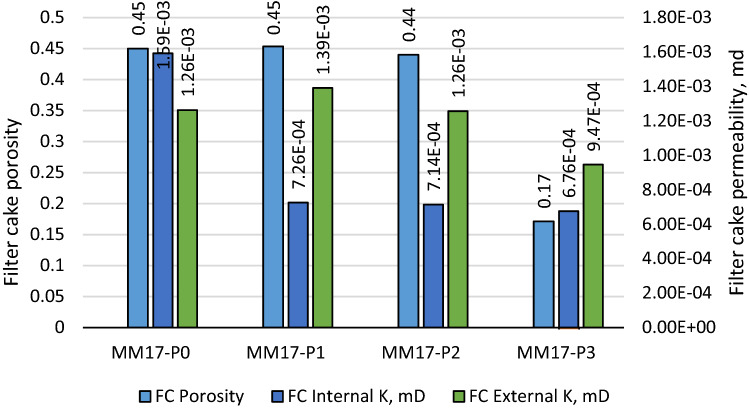
Porosity and the layer permeability of filter cake as function of perlite for mud density equal to 17.2 ppg.

Additional filter cake properties, such as porosity and permeability, were also evaluated. The porosity of the filter cake is calculated using the gravimetric method, which is based on the filter cake's dimensions, saturated and dry weights^35^. The porosity values for all the samples are shown in Fig. 6. The filter cake porosity for MM17-P0 was 0.45, and it kept almost constant for MM17-P1 and MM17-P2. It drops to 0.17 with the 3 gm. perlite addition for MM17-P3 The filter cake permeability is calculated using Lee model^35^, which depends on the filtration rate per area (q), the filter cake thickness, ($$T{h}_{fc}$$), filtration viscosity ($$\mu $$), and the differential pressure across the filter cake used during the experiment ($${p}_{fc})$$. The filter cake permeability ($${K}_{fc}$$) can be estimated as the following:

1 $$\begin{array}{c}{K}_{fc}=14700 \frac{q* {Th}_{fc}*\mu }{{p}_{fc}}\end{array}$$

The permeability values for the internal and external layers are shown in Fig. 6. The internal filter cake permeability for MM17-P0, MM17-P1, MM17-P2, and MM17-P3 are 1.59E−3 md, 7.26E−4 md, 7.41E−4 md, and 6.76E−4 md, respectively. For the external filter cake permeability, MM17-P0 showed permeability equal to1.26E−3 md, and it stayed in the same range for MM17-P1 and MM17-P2. It drops to 9.47E−4 md for the high perlite concentration (i.e., MM17-P3).

**Figure 7:**
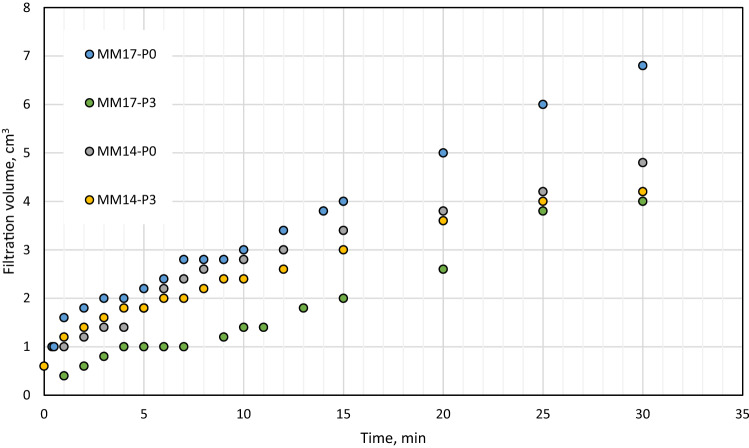
Filtration behavior as function of perlite concentration for mud samples with perlite concentrations equal to 0 and 3 lb/bbl.

**Figure 8:**
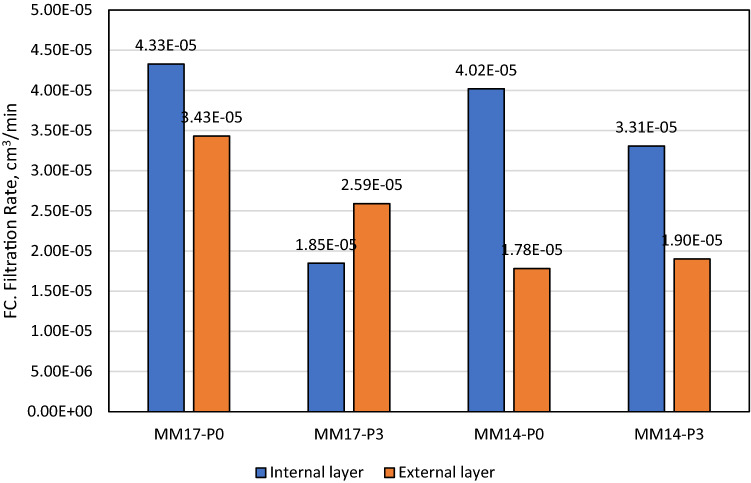
Filter cake rate for internal and external layers as function of perlite concentration for mud samples with perlite concentrations equal to 0 and 3 lb/bbl.

**Figure 9:**
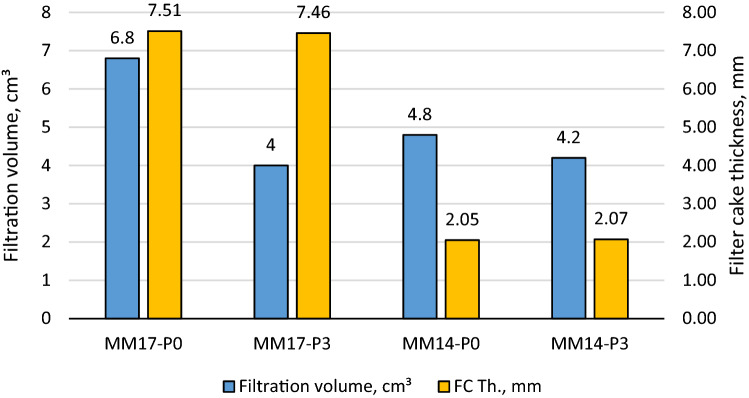
Filter cake thickness and filtration volume as function of perlite for mud samples with perlite concentrations equal to 0 and 3 lb/bbl.

**Figure 10:**
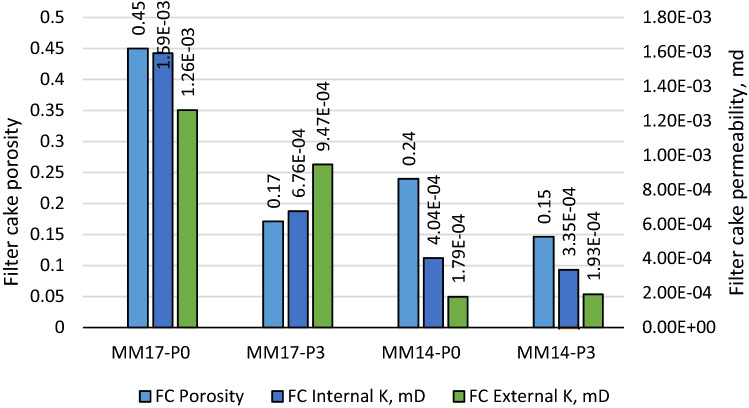
Porosity and the layer permeability of filter cake as function of perlite for mud samples with perlite concentrations equal to 0 and 3 lb/bbl.

Interestingly, MM14-P0 and MM14-P3 (i.e., mud density equal to 14.25 ppg) showed similar results in the fact that perlite improve the filtration despite the differences with the previous samples which are the mud density and the filtration medium permeability. Initially, the filtration behavior of MM14-P0 and MM14-P3 are shown in Fig. 7, they were plotted with MM17-P0 (mud density equal to 17.2 ppg with 0 lb/bbl) and MM17-P3 (mud density equal to 17.2 ppg with 3 lb/bbl) for comparison purpose which will be done for the following figures. The filtration rate changes for MM14-P0 at 5 min while MM14-P3 at 6 min compared to 7 min for MM17-P0 and MM17-P1. Commonly, the perlite particles were able of decreasing the buildup rate for both layers as shown in Fig. 8. The filter cake buildup rate decreased from 4.02E−5 to 3.31E−5 with the addition of perlite for the internal layer, and from 1.78E−5 to 1.9E−5 as perlite added for the external layer. Correspondingly, the filtration volume dropped by 12.5% (i.e., from 4.8 to 4.2 cm^3^) with the addition of perlite as shown in Fig. 9 while the filter cake thickness was not influenced by perlite. Likewise, the filter cake porosity and the permeability for both layers were affected by the perlite addition as shown in Fig. 10. The porosity decreased by 37.5% by the addition of perlite in the light mud while it reduced by 62% in the heavy mud. In similar manner, the internal filter cake permeability dropped by 17% and remain almost the same for the external filter cake permeability. The compact of the filter cake sample could be also observed visually through the formed mud cake samples as shown in the images of the filter cake (Appendix-Fig. A1).

Moreover, the plastic viscosity was slightly affected by the perlite addition in both densities, it went from 25.2 to 28.6 cP upon 3 lb perlite addition in mud with 14.3 ppg, and from 29.2 to 32.9 in 17.2 ppg. Similarly, the yield point raised from 42.3 to 44.3 in the low density and from 46.4 to 48.8 in the high density upon 3 lb of perlite added.

## Discussion

The slowness in internal filter cake builds up that is shown in Fig. 4 was attributed to the perlite particles, which was not evident as the external filter cake. Since the internal layer formed first, this can be explained as the perlite concentration in MM17-P0 and MM17-P1 was concentrated in the internal layer with low concentration in the external layer. This fact is the reason behind the huge difference between the layers buildup rates in MM17-P0 and MM17-P1. On the other hand, MM17-P2 and MM17-P3 had higher perlite concentration that was able to impact both layers. Moreover, perlite showed excellent ability in reducing the filtration volume in Micromax mud similar to other drilling fluid (i.e., barite and hematite) with 41% reduction in the filtration volume. Moreover, perlite particles were able of governing the porosity since the flake shape supports in closing the pores which results a lower porosity. However, the effect was not that high in the low perlite concentration. It became evident in MM17-P3 with almost 62% porosity reduction. In case of the filter cake permeability, by investigating the used model and link it with previous results, the most important factor is the filtration rate since the other parameters are almost constant (i.e., filter cake thickness, filtration viscosity and differential pressure). It showed the same trends exhibited in the filter cake buildup for the internal and external layers (i.e., Fig. 4). In the internal layer, perlite particles were able to reduce the permeability by 54%, 55%, and 58% for MM17-P1, MM17-P2, and MM17-P3, respectively. For the external layer, which is less impacted compared to the internal layer, perlite particles had the highest impact at MM17-P3 with 25% reduction in the permeability compared to MM17-P0.

Excitingly, the filter cake buildup rate for internal and external layers for MM14-P0 and MM14-P3 showed reverse trend compared to high density mud formulations as shown in Fig. 8. Adding perlite particles to the high-density mud samples was able of decreasing tremendously the buildup of the filter cake internal layer where it became lower than the buildup rate of the external layer. However, this is not the case in the low-density mud where the external layer still has lower buildup rate after the perlite addition. This can be attributed to the low dosage of solid particles in the low-density mud which means there are lower packing compared to the high-density mud. This will affect the distributions of the perlite particles in the drilling fluid. Clearly, the impact of perlite on the filtration volume is relatively low which is acceptable since the original filtration volume was not that high (6.8 cm^3^). Another important factor is that the filtration medium permeability, which could be the reason behind the different buildup behavior. The higher permeability corresponds to easier filtration into the core samples. Even though the different in permeability is small between the core samples for the high density mud, however, such a difference can have a minimal impact as well on the results^35^. Moreover, the filter cake thickness was not affected by the addition of perlite, and the low values of the filter cake thickness in comparison to the high-density mud can be attributed to the pore structure of the sample which was not looked into in this study^35^.

**Figure 11:**
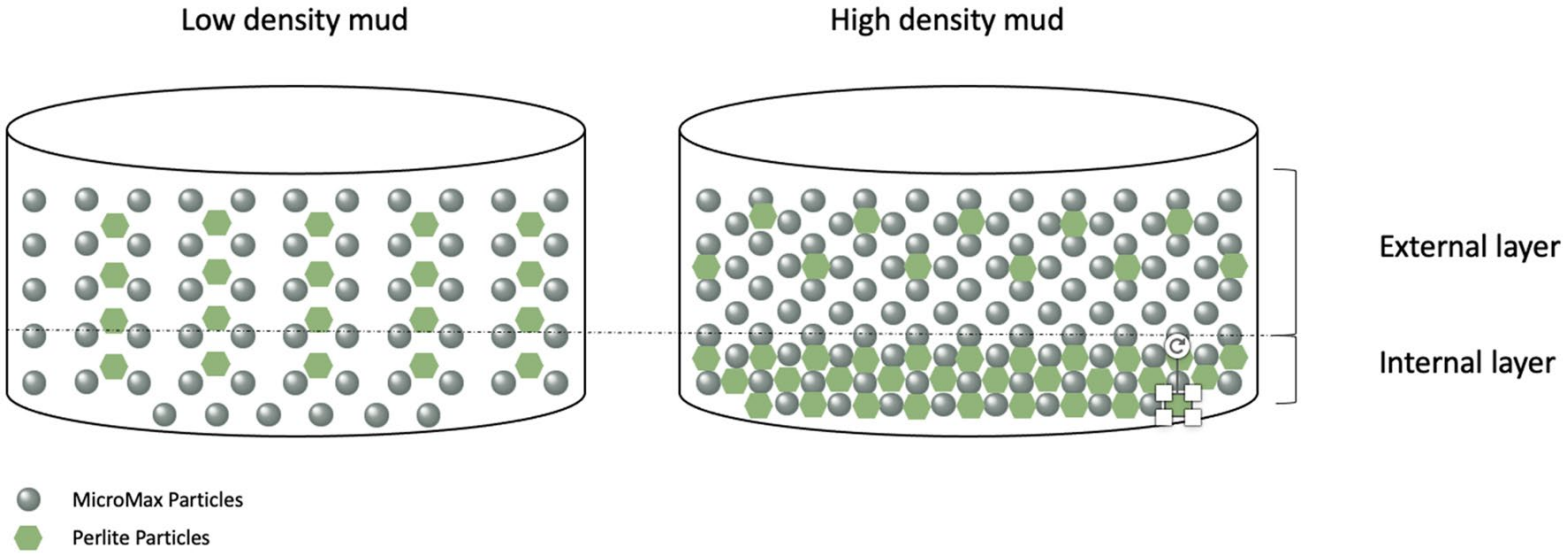
Filter cake diagrams for the low-density and high-density samples to show the perlite distribution among the filter cake.

**Figure 12:**
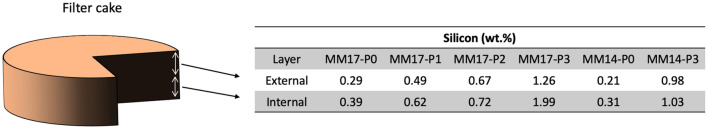
Filter cake diagrams and the corresponding Si wt% for the external and internal filter cake layers in each mud samples.

Figure 11 illustrates how the perlite particles might acted in the low- and high-density mud. Due to the low packing in the low-density mud, there was a large space for perlite particles. Such a space prevented the perlite particles at the used concentration (i.e., 3 lb/bbl) from having a significant influence on the permeability and porosity of the filter cake. On the other hand, the high-density mud has high packing due to the high dosage of solids, this implies there is a smaller space in comparison to the low-density mud for the perlite particles. Hence, there is a high probability for the perlite particles to closes the gaps between the solid particles which affect positively (i.e., lower permeability and porosity) the properties of the filter cake as observed by this work result. The energy dispersive spectroscopy in the scanning electron microscopy (EDS-SEM) confirmed the distribution of perlite particles in the filter cake. It is an elemental analysis of the sample surface; it can be used to identify the elements on the sample surface. Since the main difference between the mud formulations used in this work is the perlite concentration and it consist mainly of silicon. The perlite distribution along the thickness of the formed filter cakes was measured. Figure 12 shows the percentage of silicon in each filter cake layer for each mud formation samples. Even though the percentages of the silicon are extremely small, this is due to the fact that the perlite as ratio compared to the entire formulation is very small. The manganese element covers almost 80% of the EDS reading due to the high concentration of Micromax in the formulation. The process of selecting the line was performed by checking different location in the sample with highest silicon concentrations, then selecting the same line length percentage across the different samples to have fair comparison. It is worth mentioning that these concentrations are shown in small area in the filter cake, but it was assumed that the line represents the whole filter cake. The external layer for MM17-P0 showed 0.29% and the internal layer had 0.39% of silicon and MM14-P0 had 0.21 and 0.31 for the external and internal layers, respectively. The sources for silicon in the used drilling formulation are bentonite and perlite, bentonite concentration was fixed, and the perlite was varied, hence silicon percentage in the first and fifth samples is attributed to the bentonite and the additional change in silicon concentration in the other samples can be attributed to the perlite particles. MM17-P1 showed higher silicon concentration in both the external and internal layer which equal to 0.49% and 0.62%, respectively. The perlite concertation increased in MM17-P2 which reached to 0.67% and 0.72% for the external and the internal layers, respectively. Interestingly, the perlite concentration jumped in MM17-P3 to 1.26% and 1.99% for the external and the internal layers, respectively. Similar results were found in sanple-6 but with different trends which agrees with previous founding. Perlite concentration in the external layer equal to 0.98% and the internal layer equal to 1.03%. For the rheological properties, the perlite assisted in increasing the plastic viscosity and yield point as shown in the previous section.

## Conclusions

Filtration agent is an important additive in the drilling fluid, optimizing the filtration agent is the key factor for improving the filter cake characteristics and the filtration behavior. At two different packing doses, the filtration and filter cake features of manganese tetroxide water-based drilling fluid were examined in this study. Perlite particles were added with different concentrations to evaluate its ability, as a filtration agent, for improving Micromax mud cake, and the following conclusions can be made;

1. Perlite was able to improve the filtration and filter cake properties for high packing mud significantly (i.e., density equal to 17.2 ppg), the optimum concentration was 3 lb/bbl.
2. The filtration volume, filter cake porosity, filter cake internal and external layers permeability were decreased by 41%, 62%, 58%, and 25%, respectively.
3. In the low packing mud, the 3 lb/bbl was studied and it was capable to improve slightly the filtration characteristics from the based drilling mud. It reduced the filtration volume by 12.5%, filter cake porosity by 39%, and the filter cake internal layer by 17%. The filter cake external layers permeability remined unchanged.
4. Perlite particles were concentrated in the internal layer for high packing mud, while they were distributed somehow evenly in low packing mud formulations.
5. Perlite exhibited the ability for increasing the plastic viscosity and yield point in both low and high drilling fluid densities within the acceptable range.

## Supplementary Information

Supplementary Figures.

## Data Availability

All data generated or analysed during this study are included in this published article.
