# Supplementary material for: Improving the filtration properties for manganese tetroxide mud utilizing perlite particles to drill wide-range permeability sandstone formation
Source: Sci Rep. 2022 Nov 2;12:18445. doi: 10.1038/s41598-022-21897-8 (PMC9630278; doi:10.1038/s41598-022-21897-8)
Supplement: Supplementary file 1 — Supplementary Figures. [file 41598_2022_21897_MOESM1_ESM.docx]

# Appendix:


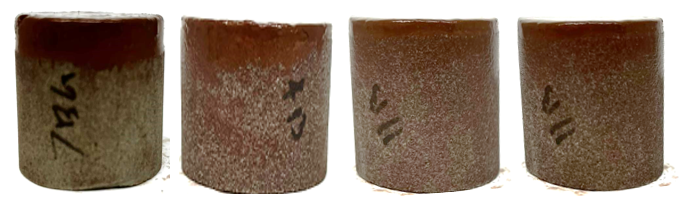


Direction of increasing the perlite concentration

Figure A1: Filter cake samples as function of adding the perlite particles
